# Contribution of diffusion-weighted imaging to conventional MRI for detection of haemorrhagic infarction in ovary torsion

**DOI:** 10.1186/s12880-017-0232-6

**Published:** 2017-11-22

**Authors:** Oğuzhan Özdemir, Yavuz Metin, Nurgül Orhan Metin, Ali Küpeli

**Affiliations:** 1 Faculty of Medicine, Department of Radiology, RTEÜ, 53100 Rize, Turkey; 2 Department of Radiology, Muş State Hospital, Muş, Turkey

**Keywords:** Diffusion-weighted imaging, Haemorrhagic ovary torsion, Magnetic resonance imaging

## Abstract

**Background:**

To assess the role of DWI in differentiation haemorrhagic ovary infarction from non-haemorrhagic one.

**Methods:**

For this prospectively designed study, of 117 female patients who presented with acute lower quadrant pain and underwent MRI for suspicion of ovary torsion, results of only 29 patients (mean age, 24.7; SD, ±5.7; age range, 18–37), with surgical and pathological confirmation of adnexal torsion, were included to the study. All patients underwent DWI after conventional MRI. Quantitative and qualitative analysis of both the torsed and contralateral normal ovary were performed. Results of conventional MRI and DWI were noted.

**Results:**

At operation 15 patients were found to have haemorrhagic infarction while 14 had non-haemorrhagic infarction. Of the 29 patients, 17 torsed ovaries could be salvaged in a viable state. We found statistically significant correlation of the ADC values, between haemorrhagic and non-haemorrhagic ovary infarction. The ADC values were significantly lower in patients with haemorrhagic infarction than non-haemorrhagic ones (*p* < 0.001). Using an ADC threshold of 1.27, the sensitivity of DWI for haemorrhagic infarction was 0.93 and specificity 0.85.

**Conclusion:**

DWI may be used with a significant success for the preoperative diagnosis of haemorrhagic infarction. This may be alerting for pre-emptive surgery in avoiding serious complications and preventing irreversible structural damage of the ovary.

## Background

Ovarian torsion results from a partial or complete rotation of an ovary or ovarian vascular pedicle about its long axis [1]. The rotation causes a circulatory stasis that is initially venous but becomes arterial with progression of the torsion. Complete torsion results in obstruction of the arterial blood supply, causing gangrenous, haemorrhagic infarction [2]. The clinical presentation is quite variable and includes sudden onset of severe pain, vomiting, nausea, leukocytosis, fever, and palpable adnexal masses [3, 4]. There are many differential diagnoses with the potential to mimic ovary torsion, so the correct diagnosis is quite difficult, even in experienced hands, despite the multimodality imaging tools available.

Ovarian torsion is a surgical emergency because a delay in diagnosis and surgery may lead to necrosis and loss of ovarian viability and thus to significant morbidity and even mortality. It is reported that, despite significant progress in imaging technology, salvage surgery is still not possible in a great number of patients, due to delays in diagnosis [5–7]. Although detorsion with surgery may allow salvage of a viable ovary without critical complications, the salvage rate is reported to be below 10% in adults but as high as 27% in children [8]. Therefore, preoperative diagnosis of haemorrhagic infarction associated with serious complications and loss of ovarian viability may assist with decisions about pre-emptive surgery.

Ultrasonography (US) is the first-step imaging modality, but its sensitivity and specificity is not sufficiently high. Although US gives information regarding torsion, such as an ovarian enlargement, diminished or absent blood flow (by Doppler mode) and presence of a mass, it does not give information about the viability of ovarian tissue so as to rule out haemorrhagic infarction [9, 10].

Conventional magnetic resonance imaging (MRI) is an efficient imaging modality in the diagnosis of ovarian torsion. The imaging findings are ovarian enlargement and Fallopian tube thickening, wall thickening of the torsed adnexal cystic mass, ascites, uterine deviation to the torsed side, adnexial haemorrhage and lack of contrast enhancement of the adnexal mass [11–13].

Diffusion-weighted imaging (DWI) is now an accepted imaging method in body imaging for the detection and characterisation of focal lesions [14–18]. It is reported that following ovary torsion, ischemic damage to the sodium-potassium pump occurs that leads to deterioration of the water flow between the intracellular and extracellular compartments. DWI allows a quantitative evaluation of molecular diffusion caused by Brownian motion and is proven to have a high sensitivity enabling the early detection of cytotoxic oedema and haemorrhage. DWI provides clearly a contrast between ischemic and normal tissue, and ADC maps show the restricted diffusion numerically [19]. Furthermore, in the clinical practice of neuroradiology, it is well known that blood products with different stages have magnetic susceptibility effect on DWI. DWI in cerebral parenchymal haemorrhagia reveals a strong hyperintense signal in hyperacute and late subacute stages, and a strong hypointense signal in acute and early subacute stages. ADC values in hyperacute, acute and early subacute stages of parenchymal haemorrhagia as well as haemorrhagic infarcts were reported to be clearly lower than non-haemorrhagic ischemic stroke [20, 21].

This study, therefore, hypothesises that DWI may improve the diagnostic efficacy of haemorrhagic infarction in ovary torsion when combined with conventional MRI. Accordingly, the goal of this prospective study was to evaluate the value of DWI in respect of the following points: first, to discriminate haemorrhagic infarction from non-haemorrhagic infarction, and hence to assess the viability of ovarian stroma, and second, to investigate whether it (DWI) has any impact on management. To our knowledge, this study is the first prospectively designed study to research the efficacy of DWI for differential diagnosis of haemorrhagic and non-haemorrhagic infarction as well as distinguishing torsed ovary from contralateral non-affected ovary.

## Methods

### Patient selection and inclusion criteria

**Fig. 1:**
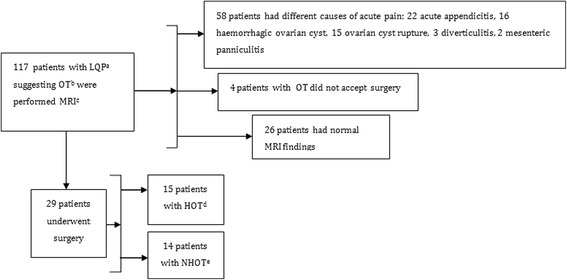
Patient flow diagram

From a total of 117 female patients presenting with acute lower quadrant pain with suspicion of ovarian torsion who underwent MRI between August 2013 and December 2016, 29 patients (mean age, 24.7; SD, ±5.7; age range, 18–37) with final surgical and pathological proof of adnexial torsion were included in this prospective study. Pregnant patients and those under the age of 18 were excluded. The study flow diagram is shown in Fig. 1. All patients underwent DWI after conventional MRI. The age, clinical characteristics and laboratory findings of the patients were noted. This study was approved by the Institutional Review Board of our university hospital and written consent acquired from each patient.

### Imaging protocol

The MRI studies were performed with a 1.5-T MRI scanner (Magnetom® Aera; Siemens, Erlangen, Germany) using an eight-channel phased array coil.

For the conventional MRI, coronal half-Fourier acquisition single-shot turbo spin-echo (HASTE) images (repetition time (TR)/echo time (TE), 1000/89; slice thickness, 3 mm without gap) were initially acquired for an overview. Subsequently, axial and sagittal T2-weighted fast spin echo (FSE) images (TR/TE, 3700–4500/85–102 ms; echo train length (ETL), 18; slice thickness, 5 mm; interslice gap, 1.5 mm; matrix, 256 × 256; field of view (FOV), 400 mm; number of signal averages (NEX), 4; acquisition time, 3 min), and axial and/or sagital T1-weighted GRE images with fat suppression (TR/TE/Flip angle (FA), 140–404/4–4.03 ms/70–75°; slice thickness, 4 mm; interslice gap, 1.5 mm; matrix, 256 × 256; field of view (FOV), 400 mm; number of signal averages (NEX), 4; acquisition time, 1 min) were acquired. Finally, the contrast-enhanced imaging was performed, using the following parameters: axial and/or sagittal GRE T1-weighted images with fat suppression (TR/TE/FA 140–409/4–4.73 ms/70–75°; slice thickness, 4 mm; interslice gap, 1.5 mm; matrix, 256 × 256; FOV, 400 mm; number of signal averages (NEX), 4; acquisition time, 1 min). All the conventional MRI images were obtained with free-breathing.

The DWI parameters were as follows: axial diffusion-weighted single-shot echoplanar sequence (EPI) with SPAIR fat suppression technique, with free-breathing (TR/TE, 7500/62–80 ms; matrix, 192 × 192; slice thickness, 5 mm; interslice gap, 2 mm; bandwidth, 1269 Hz/pixel; NEX, 6; FOV, 400 mm; PAT factor, 2; PAT mode, generalised autocalibrating partially parallel acquisitions (GRAPPA); acquisition time, 3 min; b values, 0, 500 and 1000 s/mm^2^).

### Image analysis

All the patients had clinical suspicion of ovary torsion at presentation. They were initially imaged with US, and those with findings of ovarian torsion or uncertain ovarian pathology on US as well as those without any US findings but still with suspicion of ovarian torsion were then performed MRI. Two experienced abdominal radiologists (10 and 5 years) prospectively interpreted the MRI of these 29 patients. Initially they reviewed the conventional MR images, and shortly after (during the same period of initial review), they made an assessment with the addition of the diffusion-weighted images. All interpretations were made by consensus, reached immediately or, where necessary, by collaborative discussion. The readers were aware of the clinical suspicion of ovary torsion and the laboratory findings.

On conventional MRI, the reviewers looked up for a swollen ovary, the diameter of both ovaries, Fallopian tube thickening, presence of twisted pedicle sign, peripherally displaced follicles, characteristics of possible mass, free peritoneal fluid, hemoperitoneum and ovarian stromal haemorrhage (for haemorrhagic infarction [19]. For haemorrhagic infarction discrimination, the reviewers performed a visual analysis on the conventional MRI sequences. The features considered in looking for haemorrhagic infarction were T1 hyperintensity in the stroma of swollen ovary or hypointensity on T2 images, compared to iliopsoas muscle. T1 and T2 signal changes were also analysed for hemoperitoneum in free peritoneal fluid as compared to urinary bladder. The signal changes in the case of hemoperitoneum were either T1 hyperintensity or T2 hypointensity.

The ovaries were prospectively assessed visually and quantitatively with the DWI sequences as follows:

- Visual analysis
  Visual analysis of the signal intensity was performed on b 1000 images. The signal intensity was evaluated in comparison to iliopsoas muscle as follows: hyperintense, hypointense or isointense. A strong or very strong hyperintense/hypointense signal was regarded as abnormal. As in the b 1000 images, a visual assessment was also performed on ADC maps in comparison to iliopsoas muscle as follows; hyperintense, hypointense or isointense. An iso- or hypointense signal was interpreted as abnormal. A strong hyper- or hypointense signal on the b 1000 images with concomitant strong hypointense signal on the ADC maps was considered as haemorrhagic infarction.
- Quantitative analysis
  The reviewers measured the signal intensity of ovarian stroma on T1-, T2- and diffusion-weighted imaging. The signal intensity of urine in the bladder was also measured to obtain ovarian stroma-to-urine signal intensity ratios. The ADC maps were automatically generated on the scanner console using 3 b factors (0, 500 and 1000 s/mm^2^). The ADC value was calculated with a linear regression analysis of signal intensity. For quantitative analysis on ADC map, region of interest (ROI) was placed in swollen ovary stroma. We used the images of both the conventional MRI and the DWI to predict the most suitable part into which to insert the (circular or round shaped) ROI in ADC maps. The ROI was made as large as possible, intended to cover the most hypointense parts of the swollen ovary stroma, with care taken to avoid adding any suspicious mass or follicle. At the same time, similar evaluations were made in the contralateral ovary stroma. The ROI size ranged from 0.14 to 1.95 cm^2^ (mean, 1.16 cm^2^) for the torsed ovary and 0.12 to 1.5 cm^2^ (mean, 0.97 cm^2^) for contralateral normal ovary. For each patient, the ROI was positioned at three different places, and the mean of these was used as the ADC value. The conventional MRI and DWI datasets were evaluated in the independent workstation (Syngo.via, Siemens) for post-processing and ADC map analysis.

The visual analysis of free peritoneal fluid on DWI was evaluated as follows: iso-, hyper- or hypointense compared to urinary bladder. A hypo- or hyperintense signal on the b 1000 image or strong hypointense signal on the ADC map was considered as hemoperitoneum. All patients with hemoperitoneum were confirmed at surgery.

### Statistical analysis

The data were analysed using the MedCalc package (MedCalc Statistical Software version 16.8; MedCalc Software bvba, Ostend, Belgium) and Social Sciences (SPSS 13.0 Statistical Software; SPSS Inc., Chicago, IL, USA). The means and ranges of age, long diameters of ovaries and ADC values of ovaries were calculated. A Kolmogorov-Smirnov test was used to show deviation from the normal distribution. A paired t-test was used to compare the ADC values with the diameters of the ovaries with and without a torsion. We used unpaired t-test to compare signal intensities of T1-, T2- and diffusion-weighted images. Optimal cut-off points for the ADC values for the probability of torsion were found via ROC analysis. If the obtained ADC value was less than the given cut-off value, the patient was considered to have ovarian torsion. If not, the patient was considered not to have ovarian torsion.

The same analysis was repeated for differentiation of haemorrhagic from non-haemorrhagic infarction. Sensitivity, specificity, positive predictive value (PPV), negative predictive value (NPV) and accuracy of ADC values were calculated. The area beneath the fitted binormal ROC curve (AUC) was used to measure diagnostic efficacy. McNemar’s test was used for comparison of the diagnostic performances of MRI with and without DWI in the detection of ovarian torsion, haemorrhagic infarction and hemoperitoneum. A *p* value of less than 0.05 indicated a significant difference.

## Results

Table 1 summarises conventional MRI, DWI and ADC map visual analysis of both torsed ovary and free peritoneal fluid. Results of quantitative analysis of conventional MRI sequences (T1 and T2 images), DWI and ADC values by means of signal intensity ratios are summarised at Table 2. In Table 3 MRI findings of ovary torsion, correct diagnosis number with percentages and diagnostic performances of DWI are represented.

**Table 1:** Conventional MRI, DWI and ADC map visual analysis of torsed ovary and free peritoneal fluid

SI^a^ on T1-T2/DWI^b^/ADC^c^ map (torsed Ovary)	HI^d^ (n = 15)	NHI^e^ (n = 14)	Total (*n* = 29)
Hyperintense	6 (40%)-3 (20%)/5 (33.3%)/0	0–12 (85.7%)/12 (85.7%)/1 (7.1%)	6 (20.6%)-15 (51.7%)/17 (58.6%)/1 (3.4%)
Hypointense	2 (13.3%)-4 (26.6%)/9 (60%)/14 (93.3%)	6 (42.8%)-0/0/5 (35.7%)	8 (27.5%)-4 (13.7%)/9 (31%)/19 (65.5%)
Isointense	7 (46.6%)-8 (53.3%)/ 1 (6.6%)/1 (6.6%)	8 (57.1%)-2 (14.2%)/2 (14.2%)/57.1%)	15 (51.7%)-10 (34.4%)/3 (10.3%)/9 (31%)
SI on T1-T2/DWI/ADC map (FPF^f^)	HF^g^ (*n* = 13)	NHF^h^ (n = 14)	Total (*n* = 27)
Hyperintense	8 (61.5%)-0/7 (53.8%)/0	–	8 (29.6%)-0/7 (25.9%)/0
Hypointense	0–6 (46.1%)/4 (30.7%)/11 (84.6%)	0/2 (14.2%)/0	0–6 (22.2%)/6 (22.2%)/11 (40.7%)
Isointense	5 (38.4%)-7 (53.8%)/2 (15.3%)/2 (15.3%)	14 (100%)-14 (100%)/12 (85.7%)/14 (100%)	19 (70.3%)-21 (77.7%)/14 (51.8%)/16 (59.2%)

^a^Signal intensity

^b^Diffusion-weighted imaging

^c^Apparent diffusion coefficient

^d^Haemorrhagic infarction

^e^Non-haemorrhagic infarction

^f^Free peritoneal fluid

^g^Haemorrhagic fluid

^h^Non-haemorrhagic fluid

**Table 2:** Quantitative results of cMRI*, DWI* and ADC values

	HI^a^ (n = 15)	NHI^b^ (*n* = 14)	P value
T1	1.89 ± 0.57	1.28 ± 0.23	0.056
T2	0.78 ± 0.34	0.95 ± 0.27	0.163
DWI^c^	1.50 ± 0.42	2.45 ± 0.25	0.083
ADC^d^	1.03 ± 0.56	1.77 ± 0.31	<0.001

*Values are mean of ovary stroma-to-urine signal intensity ratios

^a^Haemorrhagic infarction

^b^Non-haemorrhagic infarction

^c^Diffusion-weighted imaging

^d^Apparent diffusion coefficient

**Table 3:** MRI findings of ovary torsion, correct diagnosis number (%) and diagnostic performances of DWI

MRI findings		Patient number	HI^a^	NHI^b^	
Torsed side	Right	17 (58.6%)	13 (76.4%)	4 (23.5%)	
	Left	12 (41.4%)	7 (58.3%)	5 (41.6%)	
Twisted pedicle sign		20 (68.9%)	12 (%60)	8 (40%)	
Fallopian tube thickening		28 (96.5%)	18 (64.2)	10 (35.7%)	
Uterine deviation		14 (48.2%)	9 (64.2%)	5 (35.7%)	
Periferally deplased follicles		18 (62.1%)	8 (44.4%)	10 (55.5%)	
Free peritoneal fluid		27 (93.1%)	19 (70.3%)	8 (29.6%)	
Hemoperitoneum		11 (37.9%)	11 (100%)	–	
Diagnosis		Patient number	cMRI^c^	cMRI and DWI^d^	P Value
Ovarian torsion		29	27 (93.1%)	28 (96.5%)	0.069
Haemorrhagic infarction		15	9 (60.0%)	14 (93.3%)	<0.001
Hemoperitoneum		13	8 (61.5%)	11 (84.6%)	<0.001
DWI performances		Ovarian torsion (total)	HI	NHI	
AUC^e^		0.975	0.886	1.000	
Cutoff level ADC^f^		2.23	1.27	2.14	
Sensitivity (%)		96.5	93.3	100.0	
Specificity (%)		100.0	85.7	100.0	
PPV^g^ (%)		100.0	87.5	100.0	
NPV^h^ (%)		96.7	92.3	100.0	
Accuracy (%)		96.5	89.6	100.0	

^a^Haemorrhagic infarction

^b^Non-haemorrhagic infarction

^c^Conventional magnetic resonance imaging

^d^Diffusion-weighted imaging

^e^Area under the curve

^f^Apparent diffusion coefficient

^g^Positive predictive value

^h^Negative predictive value

In three patients (10.3%), the contralateral normal ovary could not be visualised on MRI. The clinical onset of symptoms ranged from one to five days (mean: 2.3, ±1.1). The time between MRI and surgery ranged from three to 12 h (mean: 7.3, ±2). Of the 29 surgical procedures, laparoscopic approach was employed in 20 (68.9%) and open surgery in nine (31%). Ovarian torsion occurred in the right side in 17 patients (58.6%) and in the left in 12 patients (41.3%). Haemorrhagic infarction was found in 15 (51.7%) and non-haemorrhagic infarction in 14 (48.2%) of the 29 patients, and in 17 patients (14 with non-haemorrhagic infarction and three with haemorrhagic infarction; 58.6%), the torsed ovary was able to be salvaged in a viable state. All the patients performed oophorectomy (*n* = 12) had pathological findings of haemorrhagic necrosis.

The majority (85.7%) of non-haemorrhagic infarctions were hyperintense, while haemorrhagic infarction appeared hyperintense in 33.3% and hypointense in 60% of patients on b 1000 images. According to the ADC maps, all patients except one had either a hypo- or isointense signal compared to iliopsoas muscle. The patient with a hyperintense signal on the ADC map, who had been reported as normal after the MRI review, was found to have non-haemorrhagic torsion at surgery.

**Fig. 2:**
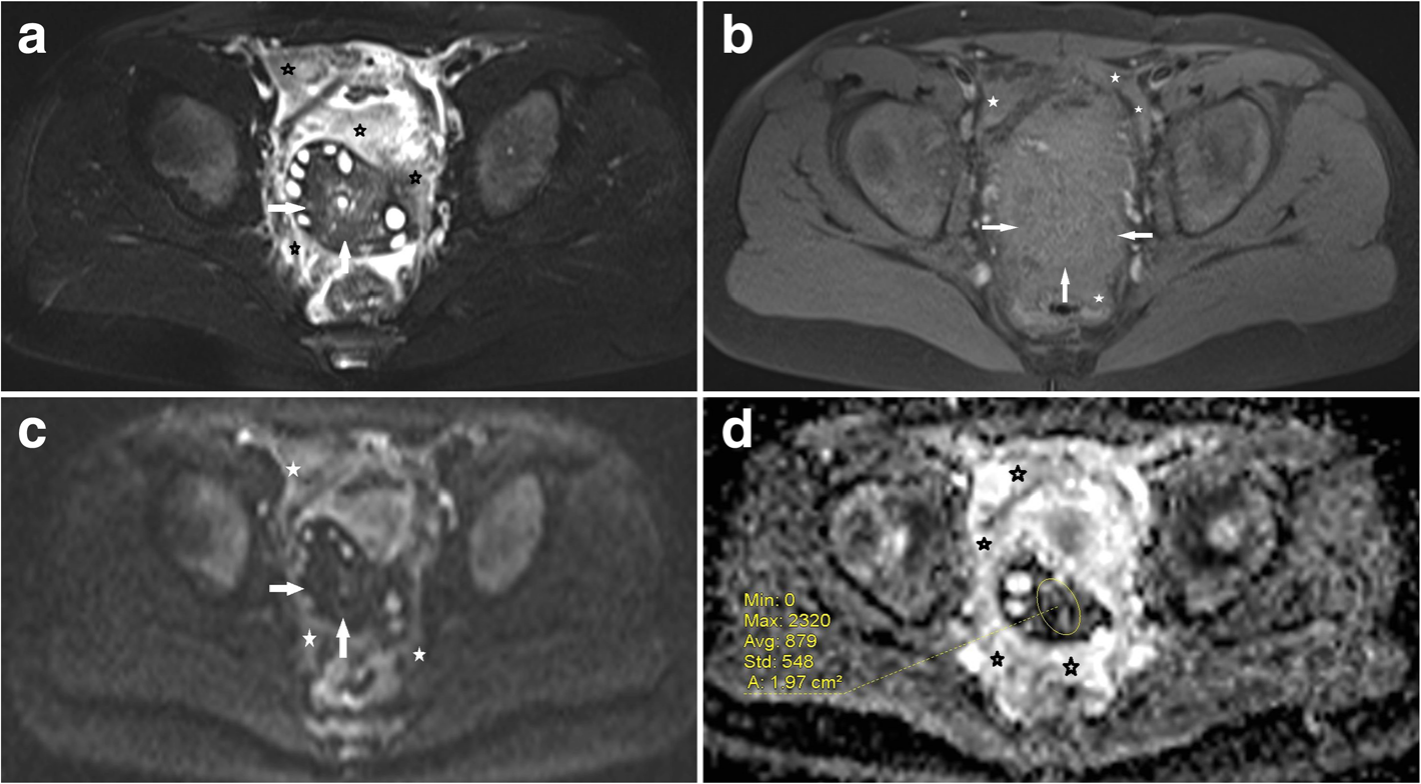
A female patient with right lower quadrant pain for three days. She had surgically confirmed haemorrhagic infarction without any space occupying lesion, and oophorectomy was performed. **a** T2-weighted fat-suppressed axial image shows an enlarged right ovary with strong stromal hypointensity (arrows) and peripherally deplased follicles. **b** T1-weighted fat-suppressed axial image reveals slight hyperintensity of swollen stroma compared to iliopsoas muscle (arrows). Note also the slightly hyperintense haemorrhagic free peritoneal fluid in the pelvic resseses (stars). **c** b 1000 image shows strong hypointensity in the ovarian stroma (arrows) and hyperintense haemorrhagic fluid in the pelvic resseses (stars). **d** Visual assessment of ADC map shows strong stromal hypointensity (ADC value, 0.87 × 10^−3^ mm^2^/s) and hypointense haemorrhagic fluid in the pelvic resseses compared to urinary bladder

**Fig. 3:**
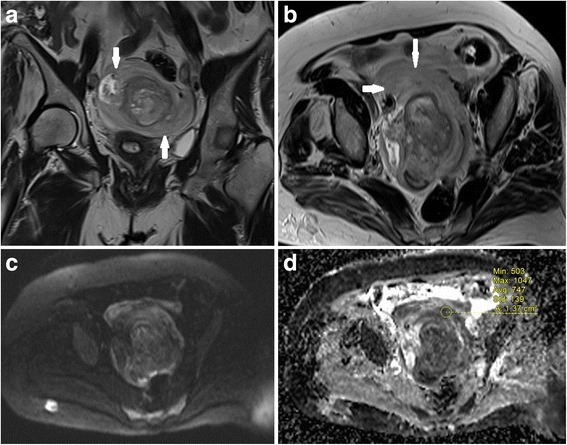
A female patient with left lower quadrant pain for 4 days. She was proved to have haemorrhagic ovary torsion due to granulosa cell tumour. **a** T2-weighted coronal MRI reveals an enlarged and oedematous left ovary (arrows) with an acompanying central mass. **b** T2-weighted axial MRI shows twisted pedicle sign (arrows) and central mass. **c** b 1000 image shows diffuse hypointensity of the enlarged ovary. **d** ADC map shows diffusion restriction of the peripheral stroma of ovary (ADC value, 0.74 × 10^−3^ mm^2^/s)

In the torsed ovary, the mean ADC value was significantly lower than that of the non-torsed ovary (mean 1.39 ± 0.58 × 10^−3^ mm^2^/s vs. 2.81 ± 0.17 × 10^−3^ mm^2^/s; *p* < 0.001). Also, the ADCs were significantly lower in patients with haemorrhagic infarction (*n* = 15, 1.03 ± 0.56 [×10^−3^ mm^2^/s]) than in those without (*n* = 14, 1.77 ± 0.31 [× 10^−3^ mm^2^/s]) (p < 0.001). The mean diameter of the torsed ovary was significantly larger than that of the non-affected ovary (mean 6.1 cm ± 0.79 vs 3.2 cm ± 0.62; p < 0.001). Using an ADC threshold of 1.27, the sensitivity of DWI for haemorrhagic torsion was 0.93 and specificity 0.85. A total of 20 patients (68.9%) had an ipsilateral accompanying mass. The histopathological diagnosis of the masses were as follows: follicular cyst (*n* = 3, simple; *n* = 4, haemorrhagic; Fig. 2), serous cystadenoma (*n* = 5), mucinous cystadenoma (n = 4), mature cystic teratoma (*n* = 2), endometrial cyst (*n* = 1) and granulosa cell tumour (n = 1; Fig. 3).

**Fig. 4:**
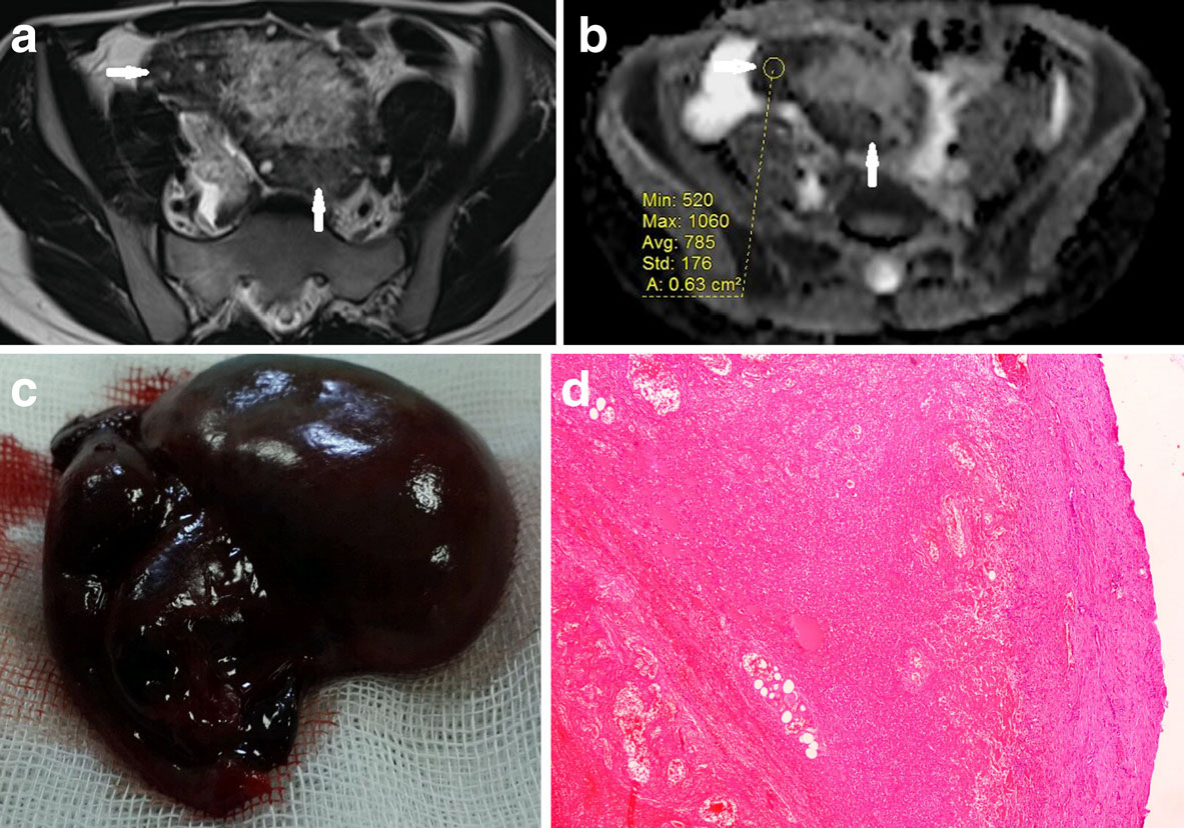
A female patient with acute left lower quadrant pain for two days. She was surgically confirmed to have haemorrhagic infarction of the left ovary without any space occupying lesion. **a** T2-weighted MRI depicts an enlarged left ovary with hypointensity of the peripheral stroma and peripherally deplased follicles (arrows). **b** ADC map shows diffusion restriction of the peripheral stroma (arrows) with a very low ADC value (0.78 × 10^−3^ mm^2^/s). **c** Gross pathological specimen shows enlarged and haemorrhagic left ovary compatible with haemorrhagic necrosis. **d** Microscopy reveals haemorrhagic necrosis

**Fig. 5:**
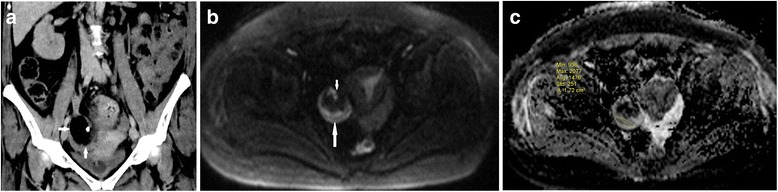
A female patient with right lower quadrant pain for three days. She had a CT scan two months ago for a different reason. At operation the right ovary was found to have slight haemorrhagic infarction on inspection and the ovary could be salvaged with partial oophorectomy. **a** Coronal CT scan depicts a right ovarian mass compatible with mature cystic teratoma (arrows). **b** b 1000 image shows an enlarged right ovary with hyperintense stroma (long arrow) containing a hypointense mass (short arrow). **c** ADC map reveals diffusion restriction (1.47 × 10^−3^ mm^2^/s) of the stroma of enlarged right ovary

Detorsion (salvage surgery) was performed in 17 patients and oophorectomy in 12 (non-salvaged). The mean ADC value was significantly higher in salvaged ovaries than that of the non-salvaged ones (mean 1.69 ± 0.47 × 10^−3^ mm^2^/s vs. 0.98 ± 0.11 × 10^−3^ mm^2^/s; *p* < 0.001). The oophorectomy patients all had a radiological report of haemorrhagic infarction. Microscopy of these patients revealed haemorrhagic necrosis (Fig. 4). Three of the patients with a diagnosis of haemorrhagic infarction on MRI were found to have slight haemorrhagia on inspection at surgery. In these three patients, it was possible to salvage the ovaries through intervention upon the decision of surgeons. The ADC values of these patients were 1.72, 1.61 and 1.30, above the ADC threshold (1.27). One of these patients had detorsion and cystectomy performed, one had detorsion and partial oophorectomy for mature cystic teratoma (Fig. 5), and the other had just detorsion. The operation times from onset of symptoms for these three patients were 29, 38 and 32 h, respectively. They became symptom-free in the following months and, thus far, have not experienced any problems regarding fertility or other serious gynaecological disease since.

There was no significant difference between conventional MRI and combined imaging (additional DWI) in the diagnosis of ovary torsion (*p* = 0.069). However, we found a statistically significant difference between these imaging methods for haemorrhagic infarction and hemoperitoneum (*p* < 0.001).

## Discussion

Studies have shown that haemorrhage in a thickened Fallopian tube, ovarian stroma or within a torsed ovarian mass and hemoperitoneum may indicate haemorrhagic infarction [6, 7]. However, conventional MRI has a low sensitivity for haemorrhagic infarction, despite its high specificity [13–16]. Our results show that conventional MRI sequences (T1 and T2) have no significant impact on differentiation between haemorrhagic versus non-haemorrhagic infarction (Table 2). This fact is compatible with the literature knowledge [10]. It is well known that torsion causes cytotoxic oedema, and hence leads to restricted diffusion on DWI in ovary stroma [19]. Meanwhile, it is a reality that blood products have magnetic susceptibility effect on DWI, as in early haemorrhagic infarction or haematomas of the brain that strongly reduces diffusion of water with the resultant low ADC values, even lower than non-haemorrhagic acute infarcts [20, 21]. Therefore, we suggest that haemorrhagic infarction of ovary may cause a further drop of ADC values than non-haemorrhagic ones, as shown in the present study. In our study, 60% of patients with haemorrhagic infarction had a hypointense signal on b 1000 images that thought to result from magnetic susceptibility effect of acute blood products. Overall, our results confirms that quantitative analysis of ADC values seems to be more reliable (*p* < 0.001) than conventional MRI sequences and DWI (b 1000 images) signal ratios in the diagnosis of haemorrhagic ovary torsion, as depicted at Table 2.

Moribata et al. [10] and Kato et al. [19] examined the contribution of DWI in the evaluation of haemorrhagic infarction in ovary torsion. Our results were compatible with the study by Kato et al. [19], in which the mean of ADC values of haemorrhagic infarction were significantly lower than of those without haemorrhagic infarction (p < 0.001). However, the mean ADC values of our study in haemorrhagic infarction and non-haemorrhagic infarction were slightly lower than in Kato et al. [19] (1.2 vs. 1.03 and 2.04 vs. 1.77, respectively). In their study, the signal intensity ratios of conventional MRI sequences were close to our results in which no significant difference was noted between haemorrhagic versus non-haemorrhagic infarction. However, we found clearly lower means of DWI signal ratios in haemorrhagic infarction as opposed to their study. This may be caused by higher number of our patients with haemorrhagic infarction as well as higher amount of acute blood products in the torsed stroma, despite measurements with same b value (1000 s/mm^2^) images. Kato et al. [19] found a 0.88 sensitivity and 1.00 specificity rate for haemorrhagic infarction using an ADC threshold of 1.80, while in our study, the sensitivity of DWI for haemorrhagic infarction was found to be 0.93 and specificity 0.85, using an ADC threshold of 1.27. This difference may be the result of the far larger patient population in our study. Another factor may be our use of three b values, as opposed to the two b values (0 and 1000 s/mm^2^) used by Kato et al. Moribata et al. [10], meanwhile, performed only qualitative analysis of signal intensities of T1 and T2-weighted and diffusion-weighted images.

Recently, Bekçi et al. [22] investigated the ADC values of torsed ovary versus non-affected ovary. They concluded that the ADC values of the torsed ovary were significantly lower than those of the non-affected ovary. They also used the ADC ratio of torsed and non-affected ovary. They suggested that the mean ratio of ADC values was significantly higher in ovary torsion than in healthy control patients and that the ADC values of the torsed ovary was significantly lower than in the non-affected ovary. Our ADC results in comparing torsed ovary versus normal ovary were similar to their results, in which the mean ADC value of torsed ovary was significantly lower than non-torsed ones (*p* < 0.001). However, our mean ADC values of both torsed and non-affected ovary were lower. This may be due to the high number of patients with haemorrhagic infarction (*n* = 15) in our study as well as the use by Bekçi et al. of only two b values (0 and 800 s/mm^2^). Actually, we do not know the exact number of patients with haemorrhagic infarction included in their study [22].

Overall, the present study differs from the above mentioned retrospective studies [10, 19, 22] in respect of the following main points: 1) our study has a prospective design, which, thus far, is unique; 2) although having similarities to the two studies cited [10, 19], we had a much larger patient group; 3) the study by Bekçi et al. [22] did not assess haemorrhagic infarction, which seems to be the most critical point of our study; 4) Moribata et al. [10] did not perform quantitative analysis of ADC values; and 5) Moribata et al. and Bekçi et al. also did not evaluate hemoperitoneum as a possible sign of haemorrhagic infarction.

Although hemoperitoneum was the least common MRI finding (37.9%) in our study, it was found to be a strong predictor of haemorrhagic infarction. We observed that most patients (86.6%) with haemorrhagic infarction had hemoperitoneum, while those without haemorrhagic infarction had no hemoperitoneum. These results were also compatible with the literature [3, 10, 19].

The timing of surgery is extremely important since a delay in operation time may result in extensive haemorrhagic necrosis and hence loss of ovarian viability [19]. We propose that after a diagnosis of ovary torsion by conventional MRI, DWI may help in differentiating between irreversible damage and reversible ischemic stroma, as shown in our study in respect of the following main points: 1) in the present study, microscopy of each oophorectomy specimen depicted irreversible damage due to haemorrhagic necrosis and radiological report of all these patients (*n* = 12) was haemorrhagic infarction; 2) most of our salvaged patients had non-haemorrhagic infarction (14 in 17, 82.3%); 3) ADC values of haemorrhagic infarction were significantly lower than non-haemorrhagic ones (*p* < 0.001); 4) ADC values of salvaged ovaries were significantly higher than non-salvaged ones (p < 0.001). Hence, it would be better to define the torsed ovaries without haemorrhagia or those with higher b values as only ischemic that may have a chance for salvage surgery. Indeed, as shown in our patients whose ovaries were laparoscopically salvaged (*n* = 17), the torsion might have result in irreversible ovarian damage and serious complications if surgery been delayed. Furthermore, our results show that a radiological diagnosis of haemorrhagic ovary torsion may be a very strong clue for irreversible ovary damage.

Our study has several limitations. Firstly, although larger than one of the comparable studies [19] and comprising the first prospective work of its kind, the study population is still relatively small. Reviewing the results of 29 patients among 117 for a prospectively designed study may lead to the idea of selection bias. However, our primary aim was to differentiate between haemorrhagic and non-haemorrhagic infarction. Secondly, although contrast agent was used in all patients, we did not analyse the contrast enhancement patterns of the torsed ovary, which is reported to aid in the diagnosis of adnexal torsion [3]. Thirdly, we did not evaluate the wall thickness of the cysts of the torsed ovary, which are reported to be thicker in haemorrhagic than in non-haemorrhagic infarction [19]. Lastly, we did not assess inter-observer variability.

## Conclusions

Diffusion-weighted imaging may assist in the preoperative diagnosis of haemorrhagic infarction and alert surgeons to the need for pre-emptive surgery to preserve ovarian viability and avoid related complications.

## Abbreviations

ADC: Apparent diffusion coefficient
CT: Computed tomography
DWI: Diffusion-weighted imaging
HOT: Haemorrhagic ovarian torsion
LQP: Lower quadrant pain
MRI: Magnetic resonance imaging
NHOT: Non-haemorrhagic ovarian torsion
OT: Ovarian torsion
ROI: Region of interest
